# Satisfaction with life during pregnancy and early motherhood in first-time mothers of advanced age: a population-based longitudinal study

**DOI:** 10.1186/1471-2393-14-86

**Published:** 2014-02-25

**Authors:** Vigdis Aasheim, Ulla Waldenström, Svein Rasmussen, Birgitte Espehaug, Erica Schytt

**Affiliations:** 1 Department of Women’s and Children’s Health, Karolinska Institutet, Stockholm, Sweden; 2 Centre for Evidence-Based Practice, Bergen University College, Bergen, Norway; 3 Centre for Clinical Research Dalarna, Falun, Sweden; 4 Institute of Clinical Medicine, Department of Obstetrics and Gynecology, Haukeland University Hospital, Bergen, Norway; 5 Møllendalsveien 6, 5009 Bergen, Norway

**Keywords:** Maternal age, Postponement of childbirth, Satisfaction with life, Primiparous

## Abstract

**Background:**

The trend to delay motherhood to the age of 30 and beyond is established in most high-income countries but relatively little is known about potential effects on maternal emotional well-being. This study investigates satisfaction with life during pregnancy and the first three years of motherhood in women expecting their first baby at an advanced and very advanced age.

**Methods:**

The study was based on the National Norwegian Mother and Child Cohort Study (MoBa) conducted by the Norwegian Institute of Public Health. Data on 18 565 nulliparous women recruited in the second trimester 1999–2008 were used. Four questionnaires were completed: at around gestational weeks 17 and 30, and at six months and three years after the birth. Medical data were retrieved from the national Medical Birth Register. Advanced age was defined as 32–37 years, very advanced age as ≥38 years and the reference group as 25–31 years. The distribution of satisfaction with life from age 25 to ≥40 years was investigated, and the mean satisfaction with life at the four time points was estimated. Logistic regression analyses based on generalised estimation equations were used to investigate associations between advanced and very advanced age and satisfaction with life when controlling for socio-demographic factors.

**Results:**

Satisfaction with life decreased from around age 28 to age 40 and beyond, when measured in gestational weeks 17 and 30, and at six months and three years after the birth. When comparing women of advanced and very advanced age with the reference group, satisfaction with life was slightly reduced in the two older age groups and most of all in women of very advanced age. Women of very advanced age had the lowest scores at all time points and this was most pronounced at three years after the birth.

**Conclusion:**

First-time mothers of advanced and very advanced age reported a slightly lower degree of satisfaction with life compared with the reference group of younger women, and the age-related effect was greatest when the child was three years of age.

## Background

The trend to delay motherhood to the age of 30 and beyond is now well established in most high-income countries [1]. In Norway the mean age of first-time mothers increased from 23 years in 1970 to 28 years in 2012 (http://www.ssb.no). Despite their declining chances to conceive, many women postpone childbearing to give priority to education and a career, for financial security, and to find the right partner [2], before trying to get pregnant. Comprehensive research has documented that this development increases the medical risks for both the mother and the infant [1,3-6]. However, studies on potential effects of advanced maternal age on emotional health are scarce. We have previously reported that psychological distress during pregnancy and postpartum was slightly more common in primiparous women of advanced age than in younger women [7]. A negative birth experience was also more common in the older women, even though they seemed to manage better than the younger, for instance with having an operative vaginal delivery [8].

In the present study we explore the association between delayed childbearing and well-being during pregnancy and early motherhood in a broader sense. A question is whether the benefit of a more stable life situation when having the baby in late reproductive life would make up for the small but increased risk of adverse medical and psychological outcomes for the individual woman. Satisfaction with life (SWL) refers to a person’s global evaluation of quality of life based on a cognitive judgment [9,10]. SWL is a measure of the life satisfaction component of subjective well-being [11]. Besides being a desirable outcome it is shown to predict future health, the quality of people’s social life and functioning [12,13], and future life events, such as divorce [14].

Due to adaptation processes and genes [15] the levels of SWL are relatively stable [16], but the degree of changes through major life events, such as pregnancy and birth, has been discussed [17]. A new mother’s SWL is not only important for her own wellbeing, but also for her baby and the rest of the family, and the risk factors to which a mother is exposed may also affect her children.

Some studies have reported that SWL increases during pregnancy [18,19] but then decreases during the first years of parenthood to pre-pregnancy level, but whether this development varies by age is unclear [20]. On the one hand, SWL would be lower in primiparous women of advanced age compared with younger women, not only because of our previous findings regarding psychological distress, but also because of the higher prevalence of adverse pregnancy outcomes, such as caesarean delivery [21], preterm birth [4,22] and infant health problems [5,6,23]. In addition to the higher prevalence in older primiparas of some socioeconomic factors [24] which also have been associated with low SWL, namely unemployment [25], financial stressors [26], and partner relationship problems [19]. On the other hand, one could assume that women who expect their first child at an advanced age are *more* satisfied with life than their younger peers because childbirth may be part of a well-defined life plan including education, career and then parenthood [24], or because women may feel more mature. One study found that SWL increased steadily during a woman’s reproductive life [17], whereas others have suggested that SWL is relatively stable over the age span [16,27], but sensitive to major life events [17-19,25,26], such as childbearing [18,19].

The aim of this study was to investigate if advanced maternal age is associated with lower satisfaction with life during pregnancy and the first three years of motherhood than a reference group of younger women, in a large population-based sample of Norwegian first-time mothers.

## Methods

### Participants and procedures

Selected data were drawn from the Norwegian Mother and Child Cohort Study (MoBa), which is a prospective population-based pregnancy cohort study conducted by the Norwegian Institute of Public Health. The MoBa study investigates socio-demographic, physical, genetic, and mental health exposure variables and outcomes in mothers, fathers and their children. The method has been described in detail in previous publications [7,28,29]. Participants were recruited from all over Norway during the period 1999–2008, and 38.5% of the invited women consented to participate. The final cohort includes 108 000 children, 90 700 mothers and 71 500 fathers. Follow-up is conducted by questionnaires at regular intervals and by linkage to national health registries. The current study is based on version 6 of the quality-assured data files, released in 2011. Informed consent was obtained from each MoBa participant upon recruitment. A postal invitation, which included an informed consent form and the first of six questionnaires, was sent out after the women had registered for a routine ultrasound examination at approximately 17 weeks of gestation. We used data from four of the questionnaires and these were completed around gestational weeks 17 and 30, and at six months and three years after the birth. A letter of reminder was sent out after 2–3 weeks in cases of unreturned questionnaires. The first questionnaire (Q1) obtained information about socio-demographic background (education, marital status, native language, income, unemployment and smoking), mother’s health during pregnancy, relationship satisfaction (a shortened version of the Relationship Satisfaction Scale) [30,31] and previous depression. In addition, the questionnaire included the Satisfaction With Life Scale (SWLS) [10,32,33](see below). The same instrument was included also in the second (Q2), the third (Q3) and the fourth questionnaire (Q4). From Q2 we retrieved data on marital status and relationship satisfaction; from Q3 marital status, relationship satisfaction; and from Q4 socio-demographic variables (marital status, smoking, financial problems), and maternal as well as infant health problems. Data on maternal age, parity, in-vitro fertilisation (IVF), mode of delivery and infant outcomes (prematurity, neonatal transfer) were retrieved from the Norwegian Medical Birth Register, which covers all births in Norway and includes information from the standardised medical records used by all antenatal clinics and delivery units in the country [34].

The present study included nulliparous women who had completed all four questionnaires, including the Satisfaction With Life Scale, and who had complete data from the Medical Birth Register on parity and maternal age. Nulliparity was defined as not having given birth previously; neither to a live nor stillborn infant after 21 weeks of pregnancy [35]. For simplicity, the term primiparity is used for women in the study, although nulliparity would have been the correct term when still pregnant. Representativeness was assessed by comparison of characteristics from a sub-sample of our study from 2003, which was approximately half-way through the data collection, with data from all primiparous women in Norway retrieved from the Medical Birth Register in 2003.

**Figure 1:**
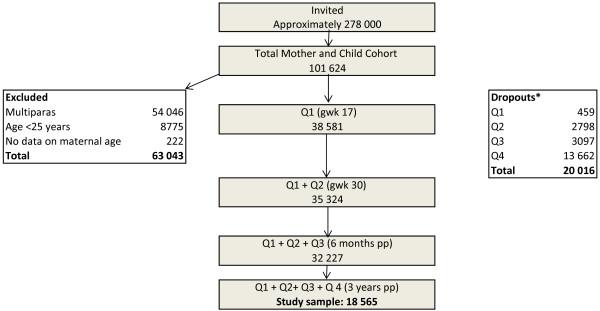
**Flow chart of women with complete data on principal outcome SWLS (n = 18 565).** Q = Questionnaire. gwk = gestational week. pp = post partum. *Dropouts = non-responders to Q2-4 (18130) or to SWLS in Q1-Q4 (n = 1886). **Medical Birth Register.

The flow chart (Figure 1) shows the initial MoBa sample and the final study group of 18 565 nulliparous women who had completed all the four questionnaires, including the SWLS. The dropouts included women who had responded to Q1 but not to one or more of the subsequent questionnaires (n = 18 130), or women who had filled in fewer than three items on the SWLS (n = 1886). Of the dropouts, 28% (n = 5635) had given birth in 2008 or 2009 and thus not yet received Q4.

### Outcome measurement

Satisfaction with life was measured by the widely used five-item version of the Satisfaction With Life Scale [10,32,33]. The responder was asked to assess the following statements: *My life is largely what I wanted it to be, My life is very good, I am satisfied with my life, I have achieved so far what is important for me in my life,* and *If I could start all over again, there is very little I would do differently*. The items were rated on a seven-point Likert scale with the following response alternatives: *totally disagree* (=1), *disagree* (=2), *slightly disagree* (=3), *neither agree nor disagree* (=4), *slightly agree* (=5), *agree* (=6), *totally agree* (=7)*,* and a summated score was calculated. The possible range of scores is from 5 (low satisfaction) to 35 (high satisfaction). Scores less than 9 indicate extremely low satisfaction with life, and scores between 20 and 24 are regarded as average [11,33]. The reliability and validity of the scale is well-established [32,33,36]. Internal consistence, measured by Cronbach’s alphas varies between 0.89 to 0.91; in the total MoBa cohort it was 0.89 in gestational week 17, 0.89 in gestational week 30, 0.89 at six months postpartum and 0.91 at three years postpartum [19]. For comparison, we retrieved similar data on SWL from the Norwegian Survey on Living Conditions 2005, including a national Norwegian sample of women (pregnant women not excluded) in the same age groups (25–31 years, 32–37 years and ≥ 38 years) [37].

### Explanatory variable

Age was defined as maternal age at the time of giving birth. There is no consensus regarding how to define ‘advanced’ or ‘very advanced’ maternal age [38] and studies use different age cut-offs [6,39,40]. In the present study, age was categorised on the basis of data from the Norwegian birth cohort from 2003, using the break point for the upper quartile (31/32 years) for advanced maternal age and the break point for the lower quartile (24/25 years) for the comparison group. To distinguish the ‘oldest’, the break point for the highest 2.5 percentile (37/38 years) was used. Consequently, advanced maternal age was defined as 32–37 years, very advanced as ≥38 years and the comparison group as 25–31 years.

### Confounders

We avoided adjusting for the natural process of ageing and therefore restricted the potential confounders to socio-demographic factors: education (9-year secondary school, 1–2 year high school, 3-year high school, University degree ≥4 years), single status, native language (Norwegian vs other than Norwegian), income (Q1) (No income, NOK <200 000, NOK 200–300 999, NOK ≥400 000), financial problems (Q4), unemployment (Q1) and smoking. To further *explore* the differences between the women of advanced or very advanced age and the reference group, we also tested the following variables in the models: previous depression, relationship satisfaction, maternal and infant health problems three years postpartum.

### Statistical analyses

For representativeness, differences in characteristics between women in the sample who gave birth in 2003 and all Norwegian primiparous from 2003 were assessed by chi-square tests. Associations between SWL in gestational weeks (gwks) 17 and 30, and at six months and three years after the birth and potential confounders were first tested by bivariate analyses using generalised linear models (GLM), and only the statistically significant confounders were retained. Multivariate logistic regression models based on the methods of generalised estimation equations (GEE) were then used to assess the association between maternal age and satisfaction with life, with adjustment for 1) time of measurement and 2) socio-demographic factors. By using GEE, we accurately dealt with the problem of possible correlation between repeated observations from the same individual and thereby obtained more precise variation estimates in the regression models [41]. We used a binary logistic model and the variance-covariance for all models was assumed to be block diagonal, but unstructured within a block defined by subjects. The results are presented as crude and adjusted mean differences with 95% confidence intervals (CI) [42]. P-values <0.05 were defined as statistically significant.

To study whether the effect of age was modified by any of the independent factors, we tested if there were interactions between age and the following factors after adjusting for all other factors: time of measurement, marital status, education, financial problems, smoking, native language other than Norwegian, relationship satisfaction, previous depression, maternal and infant health problems.

Imputations on the Satisfaction With Life Scale were made if a maximum of two of the five items on the scale were missing by using a single imputation method, the Missing Values Analyses-Expectation Maximisation algorithm [43]. As predictors, data on the remaining items on the scale were used. By imputation, 484 women could still remain in the study. Imputation was also performed on the Relationship Satisfaction Scale if a maximum of two of the five responses were missing, keeping 472 women for analysis.

The analyses were conducted using IBM SPSS Statistics version 20 (SPSS, Inc., Chicago, IL).

## Results

Table 1 shows the background characteristics of women in relation to age group. Compared with the reference group, the following characteristics were more common in women of advanced and very advanced age: high body mass index (p < 0.001), IVF pregnancy (p < 0.001), instrumental vaginal delivery (p < 0.001), caesarean section (p < 0.001), preterm birth (p < 0.001) and newborn transfer to neonatal clinic (p < 0.001). They were also more often single (p < 0.001) and high-income earners (p < 0.001). Women of very advanced age were more often unemployed (p = 0.032). Table 1 also shows that the following characteristics were underrepresented in the sub-sample of 2003 compared with women in the Norwegian birth cohort of the same year: being single, smoking, IVF pregnancy, caesarean delivery, preterm birth and neonatal transfer.

**Table 1:** Background characteristics and pregnancy outcomes in primiparous women aged 25–31 years (reference group), 32–37 years (advanced age), and ≥38 years (very advanced age), and comparison of women aged ≥25 who gave birth in 2003 in the study and in Norway in total

	**Study group (n=18 565)**			**Women ≥25 years giving birth in 2003**		
	**Age 25-31 (n=13107) %**	**Age 32-37 (n=4827) %**	**Age ≥38 (n=631) %**	**Sub-sample of the study (n=2429) %**	**Norwegian sample (n=16760) %**	**P-value**
*Sociodemographic background*						
Maternal age (years)*						0.005
25-29				55.7	54	
30-34				34.5	35.1	
35-39				9	9.6	
≥40				0.8	1.2	
Single status	2.1	3.9	10	2.8	5.7	<0.001
Native language other than Norwegian	4.9	7.5	5.7			
Unemployed	1.8	1.8	2.5			
Income (NKR)**						
<200 000	11.1	5.8	6.5			
200 000-399 999	58	46.2	45.8			
≥400 000	26	42.8	40.3			
Pregravid BMI, (kg/m^2^)						
Underweight <18.5	2.8	1.9	2.1			
Normal weight 18.5-24.9	68.2	65.4	59.6			
Overweight 25-29.9	18.9	21	22.7			
Obesity ≥30	7.7	8.6	11.6			
Smoking*	8	7.1	8.2	8	13.9	<0.001
*Obstetric and infant outcomes*						
IVF present pregnancy*	2.5	7.9	12.8	3.6	4.9	<0.001
Mode of delivery						
Vacum extraction	14.4	15.7	15.7	14.3	13.7	0.36
Forceps	2.8	3.3	3.8	2.5	22.4	0.32
Elective caesarean delivery	3.1	5.8	11.1	4.2	5.3	<0.001
Emergency caesarean delivery	9.4	12.9	19	11.2	12.5	0.03
Unspecified caesarean delivery	1.8	2.3	4	1.2	2.5	<0.001
Unassisted vaginal delivery	69	60.6	47.4	67.1	63.6	
*Infant outcomes*						
Preterm***	5.8	6.5	8.9	6.5	8.8	<0.001
Neonatal transfer	9.2	10.5	13.6	10.3	12.5	<0.001

*Data from the Norwegian Medical Birth Register.

**NKR: NOK/GBP = 10.90.

***Preterm: 22–36 weeks of pregnancy.

**Figure 2:**
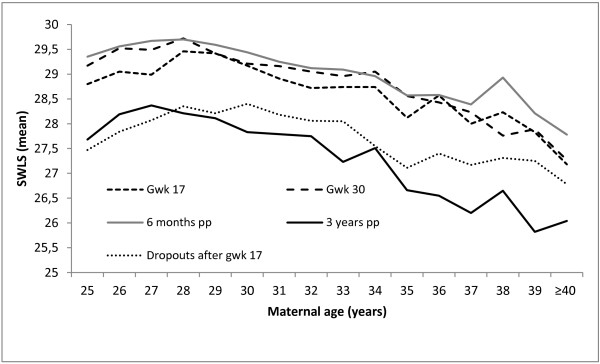
Satisfaction with life (SWLS, mean score) in gestational weeks 17 and 30 and at 6 months and 3 years after the birth in relation to maternal age in the study sample (n = 18 565), and at gestational week 17 in the dropouts (n = 5891).

Figure 2 shows the distribution of mean SWL scores by maternal age at each time point, i.e. in gestational weeks 17 and 30, and six months and three years after the birth, and also the age distribution of SWL in the dropouts in gestational week 17. During the first three time points, SWL increased from the age of 25 years to 28 years, and then decreased more or less continuously to ≥40 years. At three years after birth, the decrease started somewhat earlier, from around 27 years of age, and the decrease by age was steeper. Regardless of age, the mean SWL scores looked rather similar at the first three time points, but were much lower three years after the birth. Figure 2 also shows that women who dropped out after 17 weeks of pregnancy, and specifically the older dropouts, had lower scores than women who participated throughout the study.

Table 2 shows the mean values of SWL in the three age groups from gestational week 17 to three years after the birth, and the crude and adjusted mean differences between the women of advanced and very advanced age respectively and the reference group. SWL was slightly lower in women of advanced age (mean difference -0.64; CI 95% -0.77 - -0.51) and very advanced age (mean difference -1.57; CI 95% -1.92 - -1.21). These differences remained also after the adjustment of time and socio-demographic factors. To explore what lies behind the lower satisfaction with life in the women of advanced and very advanced age, we entered possible explanatory factors, one at the time, into the model presented in Table 2. When adding *relationship satisfaction* into the model, the mean differences between the reference group and women of advanced age (-0.5; 95% CI -0.62 – -0.38) and very advanced age (-1.18; 95% CI -1.49 – -0.86) were slightly reduced. The following factors also affected the results, but only marginally: *previous depression* (advanced age: -0.57; 95% CI -0.69 – -0.44; very advanced age: -1.41; 95% CI -1.76 – -1.07)*,* the *child’s overall health*; (advanced age: -0.63; 95% CI -0.76 – -0.51; very advanced age: -1.56; 95% CI -1.92 – -1.21), and *maternal overall health *(advanced age: -0.62; 95% CI -0.75 – -0.50; very advanced age: -1.57; 95% CI -1.92 – -1.22).

**Table 2:** Satisfaction with life by maternal age group

**Age**	**n**	**SWLS gwk 17**	**SWLS gwk 30**	**SWLS 6 months pp**	**SWLS 3 years pp**	**Mean difference**	**CI 95%**	**Adj**^**2**^	**CI 95%**	**Adj**^**2,3**^	**CI 95%**
		**Mean**	**Mean**	**Mean**	**Mean**						
25-31	13107	29.11	29.4	29.52	28.05	0		0		0	
32-37	4827	28.58	28.84	28.9	27.21	-0.6	(-0.77 - -0.51)	-0.63	(-0.76 - -0.50)	-0.7	(-0.83 - -0.58)
≥38	631	27.73	27.6	28.31	26.22	-1.6	(-1.92 - -1.21)	-1.56	(-1.92 - -1.20)	-1.32	(-1.65 - -0.99)

Mean SWLS in gestational weeks 17 and 30, and at 6 months and 3 years after the birth analysed by GEE^1^ adjusted for timepoint (Adj^2^) and sociodemographic variables (Adj^3^) with 95% Confidence Intervals (CI). Nulliparous women aged 25–31 (reference), 32–37 years and ≥38 years (n = 18 565).

^1^Generalised Estimated Equation.

^2^Adjustment for time point.

^3^Adjustment for sociodemographic variables: education, single status, native language other than Norwegian, financial problems, unemployment and smoking.

Interactions between age and all co-variates in Table 2 were investigated. Age interacted with time (p < 0.001), and the decrease by time was most obvious in women of advanced age. Age also interacted with civil status (p = 0.003), with married women of advanced and very advanced age showing lower SWL scores than the younger married women. Age did not interact with relationship satisfaction or any of the other remaining factors.

In order to validate our findings, which were based on women who participated at all four time points, we also analysed data from the larger sample of women who contributed up to the six months postpartum measurement (n = 32 227). The mean values of SWL were almost identical to those in Table 2. SWL declined slightly by age and the mean differences remained practically unchanged over the time period, also after adjusting for time and socio-demographic factors (not shown, available on request).

**Figure 3:**
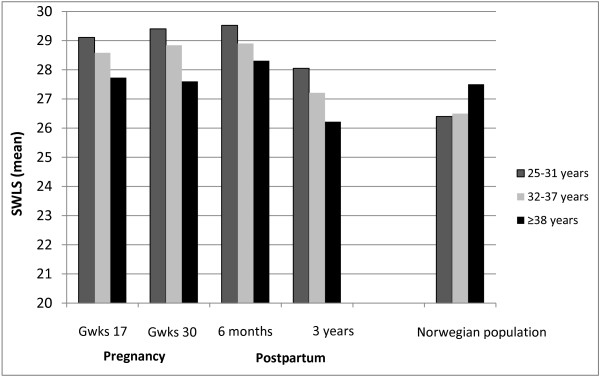
Satisfaction with life (SWLS, mean score) in nulliparous women by age group in gestational weeks 17 and 30, and at 6 months and 3 years after the birth (n = 18 565), and in a population-based sample of Norwegian women (non pregnant/pregnant) (n = 1183).

Figure 3 illustrates the mean SWL scores by age group in gestational weeks 17 and 30, and at six months and three years after birth, as well as in a population-based sample of Norwegian women (n = 1183). Overall, the level of SWL was higher in the childbearing women in our sample than in women of the same age in the total population, which included all women regardless of pregnancy, and the peak was at six months after birth. At three years after the birth, the SWL scores had decreased in the new mothers and were almost the same as in the population as a whole. Notably, SWL declined by age in the childbearing women at all four time points, but increased by age in the total population.

## Discussion

This study of pregnant and new first-time mothers showed that satisfaction with life decreased more or less continuously from around age 28 to age 40 and beyond, when measured in gestational weeks 17 and 30, and at six months and three years after the birth. When comparing age groups defined as advanced maternal age (32–37 years) and very advanced age (≥38 years) with a younger reference group (25–31 years), and taking into account the time of measurement and confounding variables, satisfaction with life was slightly reduced in the two older age groups and most so in women of very advanced age. Women of very advanced age had the lowest SWL scores at all time points and most pronounced at three years after the birth. While SWL increased in the younger women during pregnancy, the opposite occurred in the oldest. These findings suggest that the postponement of childbirth phenomenon in high-income countries may have negative effects on women’s experience of life.

The differences between the age groups were small and our results do not support the views that a more stable life situation in women who postpone childbearing weigh up for medical and psychological risks, rather the opposite. Appropriate information is needed; for women and men to make informed choices when to have children and for politicians to make it possible for young women and men to have children when it is best from a biological point of view.

One possible explanation of our findings could have been that satisfaction with life decreases by age regardless of childbirth, which would explain the lower SWL score in the oldest age groups already at the first measurement in gestational week 17. This explanation is however contradicted by the findings from the nationwide Norwegian sample of women in general, which showed that SWL was highest in the oldest women, despite the fact that the mean age in the oldest group was 39 years in our sample compared with 42 years in the population as a whole (Figure 3). Still, women who become pregnant at advanced age may have lower SWL than younger pregnant women for reasons that we could not account for in our study because of lack of sufficient information about background factors that could differ between the age groups. The effect of age was slightly reduced by controlling for relationship dissatisfaction and previous depression, both of which are more prevalent in older nulliparas [7,24] and also associated with low SWL [19,44]. One could also speculate how highly educated women, being used to control in a well-defined life plan, may have had unrealistic expectations on parenthood. The process of becoming a mother involves achieving maternal competence, adapting to changed relationships and professional goals, and a reconstruction of personal identity [45-47]. A continuous evaluation of self-image and body image against the ideal image of a mother occurs [48], which may affect confidence and self-concept [49]. Previous experiences, social support, and the baby’s behavior and condition may mediate the process [47]. The reasoning of older new mothers and their decision to tick a specific response alternative when answering the questions on SWL may therefore differ from the younger. However further research on the effect of age on parenting is needed.

The lowest SWL scores in our study were reported by the dropouts for which we had data only at 17 weeks gestation suggesting that the SWL scores in our sample would have been lower at the subsequent time points if the dropouts had been included.

Biological ageing is probably the most important explanation of our findings, due to age-related physical health problems such as fatigue and sleeping problems [24], obstetric complications [1,6,21], followed by a more negative experience of childbirth [8] and infant health problems [5,6,23]. We adjusted for both obstetric (caesarean delivery) and infant outcomes (prematurity and transfer of the newborn to neonatal infant care), as well as the following factors: IVF, fatigue and mother’s physical health during pregnancy; the mother’s physical health and breastfeeding six months after the birth; and the mother’s and the baby’s overall health three years after the birth. In spite of this adjustment, the age effects were only slightly reduced; but there might still be remaining factors on which we do not have data.

When looking at the mean SWL scores at each time point, we found that the scores were higher during pregnancy and six months after the birth than in Norwegian women in general, suggesting that pregnant women and first-time mothers in Norway are fairly happy. Having children is highly valued in Norwegian society and well supported by the welfare system. The SWL scores declined in all age groups at three years after the birth, and were then rather similar to those in the female Norwegian population. Similarly, a German panel study which reported that SWL scores increased before pregnancy and peaked just after birth, returned to the baseline level within two years postpartum [18]. However, the effect of age on SWL was most pronounced when the child was three years of age. Childrearing seemed to be most bothersome for women in their late thirties, and older mothers may also have a reduced network due to the fact that their contemporaries have finished this period in life. Lack of peer- and family support has been reported in older first time mothers from the present cohort [24] and social support is crucial to the experience of SWL [50]. Three years after the birth (when the entitled twelve months of parental leave in Norway are over), most women have started to work, suggesting that the lower SWL scores reflect the day-to-day struggle to combine the demands of parenthood and occupational work [46,51].

Our choice of age cut-off for advanced and very advanced maternal age differs from that of many other studies, but as fecundity starts to decline and medical complications increase much earlier than the more commonly used 35 years limit [3,52], the 31/32 years and 37/38 years limits were considered relevant. The non-data driven approach for age cut-off makes the decision even more valid. The strength of this study is the large population-based sample of nulliparous women including data from four time points collected over a period of 3.5 years. To our knowledge, the study is the first to investigate longitudinally the association between satisfaction with life and advanced maternal age. A limitation of the study is that women with some socioeconomic characteristics are under-represented, leading to a slight overestimation of SWL in the sample. Another limitation is the lack of SWL information before pregnancy.

## Conclusion

First-time mothers of advanced and very advanced age reported a slightly lower degree of satisfaction with life compared with the reference group of younger women, and the age-related effect was greatest when the child was three years of age.

### Ethics approval

The study was approved by the appropriate Regional Committees for Ethics in Medical Research and the Norwegian Data Inspectorate (S-97045). The Norwegian Mother and Child Cohort Study is supported by the Norwegian Ministry of Health, and the Ministry of Education and Research, NIH/NIEHS (contract no N0-ES 75558), NIH/NINDS (grant no.1 UO1 NS 047537–01, grant no 2 UO1 NS 047537-06A1), and the Norwegian Research Council/FUGE (grant no. 151918/S10).

## Competing interests

The authors declare that they have no competing interests.

## Authors’ contributions

VAA contributed to the planning of the study and analysed the data, contributed to the interpretation of findings and wrote the first draft of the manuscript. UW was the principal investigator, and contributed with the idea, the interpretation of the results and the writing of the manuscript. SR contributed in the analyses, and commented on the manuscript. BE contributed to the data analyses and the interpretation of the results. ES contributed to the planning of the study, the data analyses, the interpretation of the results and the writing of the manuscript. All authors have read and approved the final manuscript.

## Pre-publication history

The pre-publication history for this paper can be accessed here:

http://www.biomedcentral.com/1471-2393/14/86/prepub
